# The Relationship Between Individual Coping and the Need to Have and Seek Health Information Among Older Adults: Exploratory Mixed Methods Study

**DOI:** 10.2196/15858

**Published:** 2021-02-01

**Authors:** Sabine Theis, Katharina Schäfer, Dajana Schäfer, Nicole Jochems, Verena Nitsch, Alexander Mertens

**Affiliations:** 1 Institut für Arbeitswissenschaft RWTH Aachen University Aachen Germany; 2 Institut für Multimediale und Interaktive Systeme Universität zu Lübeck Lübeck Germany

**Keywords:** health, information science, systems analysis, eHealth, engineering, gerontology, information technology, mobile phone

## Abstract

**Background:**

The need to have and seek information shapes the context of computing systems. When it comes to health, individual coping influences human behavior. Therefore, the relationship between individual coping and the need to have and seek health information plays a crucial role in the development of digital health systems.

**Objective:**

This study aims to examine the relationship between individual coping and the need to have and seek health information among older adults.

**Methods:**

Questionnaires and semistructured interviews investigated the health information need (HIN) and health information–seeking behavior (HISB) in relation to the individual coping strategies of 26 older Germans.

**Results:**

The mean age of the interviewed group was 71 years (SD 7). Quantitatively, a trend was found for a negative correlation between the avoidance-oriented coping and HIN (r_s_=−0.37895; bias-corrected and accelerated bootstrap 95% BCa CI −0.730 to 0.092; *P*=.05). The qualitative results supported this finding. For some participants, information and exchange was part of dealing with their health situation, whereas others wanted to learn as little as possible to avoid a decline in their health status. The older adults acquired, collected, and exchanged paper-based health data to augment clinical information sources and support information exchange with professionals.

**Conclusions:**

Individual coping strategies are relevant for the design of digital health systems. They can support older adults in coping with their health situation, although it remains unclear how systems must be designed for people with an avoidance coping strategy to achieve the same acceptance.

## Introduction

### Background

Due to demographic changes and the underlying aging society, the number of people in need of help and care increases. At the same time, however, the number of nursing staff decreases and a gap emerges that can hardly be closed by the care provided by family members alone [1]. To address this problem, experts place great hope in health digitalization. Digital health systems offer an opportunity to support and maintain the independence and self-responsibility of older people; they enable professional health services to be made more effective and family members to be relieved [2]. Therefore, an analysis of the use context is necessary and is the subject of this study.

Cognition, knowledge, and personal experiences of the user [3,4]; the working environment; and the user’s task are a few of the standard variables considered when investigating the context of information systems. However, this context can also be described by the target group’s health information need (HIN) and health information–seeking behavior (HISB) [5-9]. HISB denotes the search for health information resulting from a perceived HIN to reach a certain goal [10]. When seeking information, a user applies different sources of information that might be analogous, such as a print medium (eg, the newspaper) or a digital medium such as the internet or a smartphone app [11,12]. In fact, the HIN of older adults showed a relationship with the use of health information seeking [13]. Here, it appeared that older adults, who require more information about their health, engage more with mobile devices such as smartwatches or mobile health apps installed on tablet PCs. However, the influences on HIN seem to be manifold.

In the health care context, where an illness often relates to a stressful situation, individual coping strategies can have an influence on patients’ HISB [14-16]. In particular, the model of information-seeking behavior by Wilsons and Walsh [17] illustrates that actions or a series of actions taken to approach an unpleasant or stressful situation include or are related to HISB. For example, people who want to avoid coping with their illness feel more overwhelmed by illness-related information and less if provided by an information presentation that fits their coping strategy. Not surprisingly, van Zuuren and Wolfs [18] found that HISB is highly related to task- and problem-oriented coping strategies and that some people even perceive the information itself as a threat similar to the illness. Lower socioeconomic status, poor health, low media attentiveness, and high affective components of information seeking were associated with overload. The strongest predictors were education level and cognitive aspects of information seeking, which indicates that health information literacy skills strongly predict the overload [19].

### Research Questions

The incidence of disease increases during the course of life. In older adults, illnesses occur more frequently from the age of 50 years. Older adults thus represent an important audience for digital health systems. Although previous work described their information needs and behavior quantitatively, qualitative descriptions and relationships with individual coping are lacking. As individual coping has a particularly strong influence on a person's behavior in the context of an illness, the following research questions (RQs) investigate the information needs (RQ 1) and information seeking (RQ 2) of older adults and their relation to individual coping (RQ 3) in a qualitative and quantitative manner: RQ 1: Which HINs do older adults have?, RQ 2: How do older adults acquire the needed health information?, RQ 3: How does the coping of older adults relate to their HINs and HISB?

## Methods

### Study Design

To answer the previously mentioned RQs, a mixed methods field study was conducted [20]. Qualitative interviews allow respondents to talk at some depth, choosing their own words to describe their HIN and HISB. Questionnaires then quantitatively measured HIN, HISB, and individual coping (Coping Inventory of Stressful Situations [CISS]).

### Participants

The sample consisted of 26 older adults living in the German state of North Rhine-Westphalia. A total of 18 interviewees also answered the questionnaires. Moreover, 8 participants answered the questionnaires only, as they refused to be interviewed directly. A total of 3 interview recordings (ID01, ID11, and ID12) were lost because of recording issues.

A total of 33% (5/15) of the interviewed participants who answered the questionnaire were male and 67% (10/15) were female. The mean age of the interviewed group was 71 years (SD 7). The participants had a rather varied level of education: 14 had completed secondary modern school (*Volksschule/Hauptschule* in German). Five of these had subsequently undergone vocational training (*Berufsausbildung*). A total of 3 participants attended secondary school (*Realschule*) and high school (*Gymnasium*), whereas only 1 participant had a university degree (*Hochschulabschluss*). Participants ID04 and ID05 were a couple and interviewed together.

All 26 participants were born in Germany. Participants were primarily office employees and craftsmen. In total, 8 of the participants who were interviewed and answered the questionnaires had a leadership position, whereas 7 did not have a leadership position. A total of 54% (14/26) were living at home with their partner, wife, or husband. The other 31% (8/26) lived alone at home, and 15% (4/26) lived at retirement homes (Table 1).

**Table 1:** Demographics (N=26).

ID	Age (years)	Gender	Educational level	Living situation	Interview
01	88	Female	Secondary modern school	At retirement home	Lost data
02	74	Male	High school	At home, with spouse	Completed
03	68	Female	Vocational training	At home, with spouse	Completed
04	80	Male	Secondary modern school	At home, with spouse	Completed
05	78	Female	Secondary modern school	At home, with spouse	Completed
06	82	Female	Secondary school	At home, alone	Completed
07	68	Female	High school	At home, alone	Completed
08	76	Female	Vocational training	At home, alone	Completed
08	77	Female	Secondary school	At home, with spouse	Completed
10	61	Male	Vocational training	At home, with spouse	Completed
11	75	Female	Secondary modern school	At home, with spouse	Lost
12	80	Male	Secondary modern school	At home, with spouse	Lost
13	64	Female	Secondary modern school	At home, alone	Completed
14	64	Female	Secondary modern school	At home, with spouse	Completed
15	66	Male	Secondary modern school	At home, with spouse	Completed
16	65	Male	Vocational training	At home, with spouse	Completed
17	65	Female	Vocational training	At home, with spouse	Completed
18	78	Female	PhD	At home, alone	Completed
19	76	Female	Secondary modern school	At home, alone	Refused
20	79	Female	Secondary modern school	At home, with spouse	Refused
21	72	Female	Secondary modern school	At home, with daughter	Refused
22	68	Female	Secondary modern school	At home, alone	Refused
22	72	Male	High school	At home, alone	Refused
24	83	Female	Secondary school	Retirement home	Refused
25	92	Male	Secondary modern school	Retirement home	Refused
26	79	Female	Secondary modern school	Retirement home	Refused

### Procedure

The interviews were conducted and the questionnaires were answered during the interviewers’ visit to the participants’ homes. During the visit, the inquiry procedure took up to 1.5 hours and started with an introduction, followed by acquiring informed consent and answering demographic questions. Subsequently, a semistructured interview was conducted, which was followed by different structured questionnaires and, finally, by the assessment of individual coping strategies via the CISS.

### Interview Guideline

The qualitative interview guideline was based on the study by Warner et al [21] (Multimedia Appendix 1), who investigated nonoccupational information needs using a framework that focused on the essential components of information needs and behaviors—the user, the needs, the sources of information, and the tools and solutions users apply—and on the interaction effects between these variables. Their tool was pretested with data from a cross-sectional and large random sample. It was adapted to the domain of personal health and shortened to the version included in Multimedia Appendix 1.

Initially, participants were queried on their required health information. Subsequently, they were asked about the information they needed during the last week or month regarding their personal health or health in general, vital data, medication prevention, treatment, and additional topics that occurred to the participants. In section 2 of the interview, participants had to rank the mentioned information needs and describe the frequency and time of occurrence of the question or the problem they had and the sources they already used, planned to use, or failed to use. In cases where no information sources were acquired, participants were asked to describe which sources they thought might have the necessary information.

### Sociodemographic Questionnaire

With this questionnaire, we queried not only standard but also theoretically relevant parameters such as age, educational achievements, professional background and available information sources, cultural background, and current living conditions (Multimedia Appendix 1).

### CISS

The CISS is a 48-item instrument used to measure 3 basic coping strategies, with 16 items per scale: task-oriented (T), emotion-oriented (E), and avoidance (A) [22,23]. Items are scored on a 5-point Likert scale (from 1=not at all to 5=very much). Higher scores indicate greater use of that particular coping strategy. To exclude the interviewee’s fatigue as an external factor, we decided to apply the *paper-and-pencil* MHS QuikScore with 21 items.

### Information Need Questionnaire

In addition to the open, semistructured interview, the need for information and the behavior were queried using a specially created, theory-based questionnaire [17]. The need for information is implicitly taken into account by the question of satisfaction with the available health information, which the participants were able to answer using a 5-point Likert scale (1=applicable, 2=rather applicable, 3=partially, 4=rather not applicable, and 5=not applicable). The corresponding questionnaire can be found in Multimedia Appendix 1.

### Qualitative Data Analysis

Theoretical thematic analysis inspired by the 6-step recursive process by Braun and Clarke was used to analyze the qualitative interview data. The main advantages of thematic analysis lie in its flexibility, usefulness, and easy access to researchers who are new to qualitative research [24]. Transcripts were analyzed thematically. Thematic analysis is characterized by an essentialist, analyst-driven, and semantic approach, which means that the coding process was done in relation to the RQs, preresearched concepts of HIN and HISB, and, thus, with regard to particular areas of interest. Progression from a semantic level to a level of interpretation gave rise to broader meanings and implications. With respect to the HIN and HISB topics, we systematically coded with the help of *Dedoose* software (University of California, Los Angeles). There were no qualitative questions on individual coping during the interviews. Individual coping was quantitatively measured using a questionnaire (CISS). The relationship between HIN and HISB and coping was analyzed by quantitatively grouping participants into the coping groups, as described in the following section, and then qualitatively describing the HIN and HISB groups.

### Quantitative Analysis

To investigate the influence of individual coping strategies on HIN and HISB of older adults, we built CISS-type clusters based on the 3 CISS dimensions: *task-oriented coping* (T), *emotion-oriented coping* (E), and *avoidance-oriented coping* (A). When a participant’s score of each of the 3 was higher than the mean of all participants on the same dimension, this dimension labeled the dimension type. This resulted in 6 CISS types—T, E, A, TE (task-emotion oriented coping), TA (task-avoidance oriented coping), and TEA (task-emotion-avoidance oriented coping)—based on which we compared the questionnaire results on information need and seeking behavior (Wilson Questionnaire given in Multimedia Appendix 1).

The descriptive analysis of the questionnaire data was carried out using Dedoose software (University of California, Los Angeles). The statistical software SPSS (IBM Corp) version 24 was used to calculate the chi-square test results attaining the relation of HIN, HISB, and CISS subscales. The CISS groups were compared qualitatively within the framework of a mixed methods analysis. An overview of the qualitative and quantitative measures and the analysis with respect to the independent variables is depicted in Table 2.

**Table 2:** Overview of the quantitative and qualitative measurement and analysis methods.

Measurement and analysis type	HIN^a^	HISB^b^	Coping×HIN/HISB
Measurement: QUAL^c^	Interview	Interview	N/A^d^
Measurement: QUAN^e^	Questionnaire	Questionnaire	Questionnaire
Analysis: QUAL	Thematic analysis	Thematic analysis	N/A
Analysis: QUAN	Descriptive statistics	Descriptive statistics	Grouping, correlation
Analysis: QUAL+QUAN	N/A	N/A	Thematic analysis of groups

^a^HIN: health information need.

^b^HISB: health information–seeking behavior.

^c^QUAL: qualitative.

^d^N/A: not applicable.

^e^QUAN: quantitative.

## Results

### HIN Interview Data

In total, 3 groups of information need emerged from the thematic analysis. The participants themselves indicated the level of HIN either directly or indirectly, for example, by naming a lot of information needs. A total of 2 independent qualitative analysts assigned them to the corresponding category. In cases where different assignments were made, the decision was discussed and then a unanimous decision was made. The following groups emerged from the analysis: (1) participants with no or low information need, (2) participants with moderate information need, and (3) participants with high information need. In addition to the intensity of information need, a thematic analysis also revealed these different topics:

1. Need concerning communication with doctors
2. Need concerning information with thematic prevention/ precaution (including questions on nutrition and sports)
3. Need for information about medication
4. Need for health-related costs and their generation
5. Need concerning information about vital data and health parameters
6. Need for better exchange between health-related actors and institutions
7. Need for information about health insurance company
8. Need for age-related possibilities for obtaining information
9. Isolated further requirements for information that could not be classified.

In the following sections, the information needs for each of the 3 groups formed, broken down by topic, are presented.

#### Group 1: No or Low HIN

This group includes ID03, ID04, ID08, ID14, and ID16. ID04 and ID16 indicated that they had no need for or did not comment on health-related information. ID14 supported the statements of ID15, but otherwise did not express its own HIN (ID15 expressed moderate HIN in topic 2). Cancer screening was an important issue for ID03. In addition, ID03 had no current HIN at the time of the interview because the participant was generally satisfied with the information transfer of his own doctors and presented it as honest, open, and to the point. The HIN of ID08 depended on their own health situation. As this was satisfactory at the time of the interview, the participant had no particular HIN. However, the question of good prevention measures is interesting for ID08. ID08 also spoke of an experience in which her doctor was unable to answer all health-related questions. She then added information from the internet to the information she received. Both ID03 and ID08 were satisfied with their health situation and showed a need for prevention/precaution (topic 2).

#### Group 2: Moderate HIN

This group includes ID05, ID06, ID10, ID15, and ID18. All interview partners, except for ID10, addressed (topic 1) a need for communication with doctors. Thus, ID05 stated that she is generally satisfied with the transfer of information between the doctor and herself. In this context, it was perceived as negative that doctors do not have or cannot take enough time for the treatment, thus leading to treatment insufficiency. ID05 commented on the medication prescription as follows:

Before that, the doctors wrote down what the heart desires. They didn’t bother at all, I think.

However, ID05 trusted the doctors. ID06 was very satisfied with the medical expertise, the related organization, and accessibility of the information she encountered. This covered the largest part of ID06’s HIN. Doctor appointments were dutifully documented by ID06:

This is very important for me. I always write down in my notebook how often I go to the doctor.

It was important for ID06 that the information comes from a doctor and that the information is bundled with this doctor. ID15 was generally satisfied with the information he gets on health-related questions:

Well, information in general has never been withheld from me when I have asked for it [both at the doctor’s office and on the internet].

Access to information was given for ID15, even if it was sometimes problematic to obtain it because of a lack of exchange between doctors. In contrast, like ID05, ID18 considered the doctors’ lack of time to be a problem:

The doctors only have three minutes’ time. How is he supposed to explain [the meaning of the diagnosis: Morbus Sudeck] to me in three minutes.

Due to the lack of time, information could not be transmitted sufficiently, which led to a compensatory measure in the form of information generation via the internet. Generally, ID18 was satisfied with the information about her health available to her. ID18 often relied on her own perception. In comparison with its sensation, ID18 ranked the quantity of information to be secondary.

ID05, ID06, ID18, and ID10, in particular, expressed an HIN on the topic of prevention/precaution (including questions on nutrition and sports; topic 2). For ID06, precaution was a very important issue, and ID18 stated that there is a need for further information. ID05, however, would have liked to get information on diet advice. ID10’s HIN focused on sports activities. For example, ID10 needed instructions for his regular sports training sequences, which he has received from video recordings on a DVD. He had a need for daily alternating sports exercises and would have liked to know how far he can increase and vary his training. In this context, ID10 had an HIN to determine his sporting progress, too:

How has body fat percentage developed over time, for example?

For ID05, ID06, and ID18, there was an HIN (topic 3) about the medication. ID06, in particular, focused on the interaction of medication. For ID06, the main trust in their own doctors was reflected in the way they deal with medication:

You don’t even want to know what can be there. We rely on the doctors.

Nevertheless, ID06 would have liked further information on medication and the reliability of medication effects, which is contradictory.

Furthermore, ID06, ID15, and ID18 had a (topic 4) need for health-related costs and how costs arise. ID15 would have liked to have more information about how hospitals and doctors bill patients. For ID18, the bills for clinical examinations and treatments were not transparent and comprehensible, which was why ID18 would have liked more information on this topic:

I do not understand the billing process, it is not comprehensible at all how they do it.

ID06 was dissatisfied with the information received regarding health insurance coverage.

ID15 expressed the need for a better exchange between health-related actors and institutions (topic 6). Thus, ID15 was dissatisfied with the information flow between doctors and believed that the views of the doctors were not sufficiently congruent. For him, this was reflected in the diagnoses that were made. The fact that doctors make different diagnoses based on the same facts ensures that the information becomes more unreliable. ID15 would have liked to see more clarity, accuracy, and congruency from doctors. Besides, he was dissatisfied with the limited and inaccurate information flow from doctors and hospitals to their patients. He mentioned the example of a planned operation that was to be performed on him:

It was already three o’clock in the afternoon, when I was supposed to be picked up and I was still lying in the bed with my hospital gown open in the back. I thought I’d get going now. And I had not received the information.

According to his own statement, after receiving no information, he checked out himself.

Moreover, ID15 would have liked more information on (topic 7) health insurance companies. Overall, ID15 was satisfied with the information he received from his health insurance company on the scope of services provided there. Accordingly, he would not consider it to be a problem that health information is being stored on his health insurance card if it was accessible to doctors, thus facilitating the exchange of information. ID15 found it interesting to know which information is stored in the health insurance card and which personal information can be viewed there.

ID18, however, had a need for age-appropriate means of obtaining information (topic 8). This was reflected in the desire for better guidance on the internet to obtain the desired information more quickly. Thematically interesting for ID18 were, among other things, hints for self-help groups to get reports of their experiences.

ID05 concluded by commenting on isolated further needs for information (topic 9). An HIN was defined here in terms of legal procedures, for example, toward companies. This was reported from a personal experience in which ID05 and ID04 became victims of a fraud during a coffee trip:

Yes, we were once badly fooled. We were on a coffee trip there and they sold us a product. [...] There were also people who said “yes, we did that too” and afterwards we found out that they mix people among the coffee trip participants who belong to them. Afterwards, you’re always smarter.

#### Group 3: High HIN

The group with high HIN encompassed ID02, ID07, ID09, ID13, and ID17. ID02, ID07, and ID17 had (topic 1) a need for communication with doctors. ID02 had the desire for more transparency in medical examinations. He was bothered by the fact that information only came when it was requested. In contrast, ID17 had a basic need for information regarding his or her health situation:

And I am someone, I said from the beginning, who wants to know what I have. I want to know how I have to handle it.

For her, this handling of information was part of their information behavior. Furthermore, the personal relationship with the doctor was important for ID17, who was also the most important information source for her.

ID07, however, reported a recent experience that has had a lasting influence:

[My daughter] who is 27 weeks pregnant, will have twins, and her gynecologist said that she has to go to her family doctor. The family doctor said that the leukocytes were too high. And he, who then sent her home, said, “I’ll check it out” and talked to her on the machine this morning and told her “yes, her gynecologist would get in touch with her next week.”

ID07 believed that doctors often lack the feeling for the context or the empathy for the patients’ situation. She firmly believes that patients, especially her own daughter, have to put up with long waiting times and are informed relatively late about their own symptoms.

ID07 described the idea of the doctors’ lack of empathy with the fact that doctors often have no intuition for someone not wanting further information. She talked about a procedure in which the flow of information led to her feeling nervous and restless:

That already strained me with what they said. “We’ll saw your bone through there,” and so on.

She also described a third incident that had a lasting impact on her information needs. ID07 was much younger, and although she was still breastfeeding, this could have been dangerous for the child, as her treating physician had prescribed cortisone (Cortisone is a pregnane [21-carbon] steroid hormone. It is one of the main hormones released by the adrenal gland in response to stress).

ID07 considered this a wrong decision that originated from a lack of information generation:

Yes, and he sees that I have a child and does not ask me if I am breastfeeding or something like that, but prescribes me a cortisone medicine. You can’t do that. That goes into the blood and then into the child.

The described incidents led ID07 to the statement that she is not a doctor’s friend and that she critically questions the information provided by them. Accordingly, she wanted several expert opinions on a diagnosis:

Somehow, I always have the feeling that I am missing information, because I say “yes okay, then I go to the next doctor. I’ll ask his opinion about that. Or I’ll ask a third doctor about this.” That is, with one piece of information, I am therefore probably not so satisfied. Probably this will not be enough for me, then I would need a little bit more.

Overall, the desire for credible doctors prevailed at ID07, as did the desire for self-determination:

You are sent from one doctor to another and don’t have much of a choice to say: “But I’m going to see another doctor.”

ID02 and ID03 commented on (topic 2) the HIN on prevention/precaution (including questions on nutrition and sports). ID02 had an in-depth interest in cancer screening. ID13, in this regard, had an HIN on pain causes and management, as she was currently in pain. In connection with this, ID13 had an HIN that deals with muscle activity and performance. She was interested in how you can plan your daily activities meaningfully in that context.

ID13 and ID17 had (topic 3) an HIN about the medication. Thus, ID13 had an HIN on the effects and intake of medication. ID13 consolidated her own doctors to obtain information. ID17 showed a need for transparent communication in the field of medication. She found it difficult that she had to act as an information source when she visits a new doctor and had to inform him about the medication she was taking. ID02 and ID13 showed a need for (topic 4) health-related costs and their generation. For ID13, medical bills and the handling of costs by doctors and health insurance companies were mentioned as interesting points. ID02 was interested in questions that dealt with the composition of treatment costs. For example, ID02 stated that the flow of information between doctors and the patient on this subject was impersonal and inaccurate. For him, it was important to be able to understand the costs.

ID02, ID07, ID13, and ID17 commented on (topic 5) an HIN on vital data and health parameters. Thus, ID02 had the desire for direct clarification of available health data, for example, measured values. ID17 showed an HIN on health-related parameters and values and their personal significance. ID17 would have liked assistance in interpreting health-related data, as described in the first case of her pregnant daughter. ID13 indicated that vital signs were generally not very important to her. Blood pressure was excluded from this, even if ID13 stated that she was able to assess it well on the basis of body sensation:

I consider it very important, but I don’t need to measure it, I can tell you by heart what it is like. [...]My blood pressure is always 140 over 80 with medication intake. [...] As soon as the lower value rises, I feel as if I really wanted to squabble.

ID17 had a basic HIN; however, ID17 did not want to be reminded of her illness every day. This included, for example, the daily wearing of a measuring device, which she considered to be very stressful for her. Her wish for information and the desire not to be constantly reminded of her illness was somewhat of a dilemma.

ID02 and ID07 had a (topic 6) need for better exchange between health-related actors and institutions. For ID07, the flow of information between doctors and patients in this field was impersonal and inaccurate. She assumed that these processes were carried out by a third party, for example, a secretary, and that the attending physician was not even informed about the costs involved. In ID07’s view, this matter was also an incomplete communication between doctors, which annoyed her personally.

On (topic 7) an HIN about health insurance companies, ID07 stated that it is a difficult matter on which she would have liked to have more information. In addition, ID13 stated that, according to her, health insurance companies work against, not with and for, patients, which leads to the exclusion of patients:

Something could come from the insurance company to make life easier for you... So that they’ll be more active in approaching people.

ID13 also expressed her opinion on (topic 8) the need for age-related means of finding information and would have liked to have easier access to it. The background was that, according to her statement, many older people do not have internet access.

Finally, ID07 and ID17 (topic 9) indicated isolated needs for further information. In ID07, it represented a desire for self-determination:

You are sent from one doctor to another and don’t have much of a choice to say: I’m going to see another doctor.

This led to an HIN about the availability of alternative doctors and a need for general information about doctors before a doctor becomes a patient. ID17 focused on the social environment and the HIN for relief measures. The reason for this need was her own heart disease manifested in the form of several heart attacks. According to patient ID09, there was an HIN for follow-up and preparation in addition to the discussion with the doctor. The doctor was considered to be the most important source of information for personal health information; however, ID09 felt that this is missing because of time pressure of the physician, lack of interest in the patient by the physician, or the fact that the physician does not take patients seriously:

They don't take you for full [...]I didn't understand at all what he said to me [...]I've written down the words (technical terms/unintelligible words), and I'm going to the family so a family member can translate them for me.

For an appropriate exchange of information about health information, ID09 required the fulfillment of emotional and interpersonal needs by the physician as a prerequisite for exchanging information in a personal conversation. If a doctor did not comply, the need for information was covered by another source of information. Most importantly, she saw a difference between the specialists and the family doctor.

### HIN Questionnaire Data

The descriptive results of the questionnaire data revealed that the information needs of older adults were quite low: 46% (12/26) of the sample were satisfied or rather satisfied 12% (3/26), 8% (2/26) were rather dissatisfied with the available health information, and 35% (9/26) were partly dissatisfied. HIN is indicated by the satisfaction with the information at hand. Information need was thus measured by how applicable participants considered the statement “*I am satisfied with the information I have available on health/my health*.”

### Information-Seeking Behavior Interview Data

**Figure 1:**
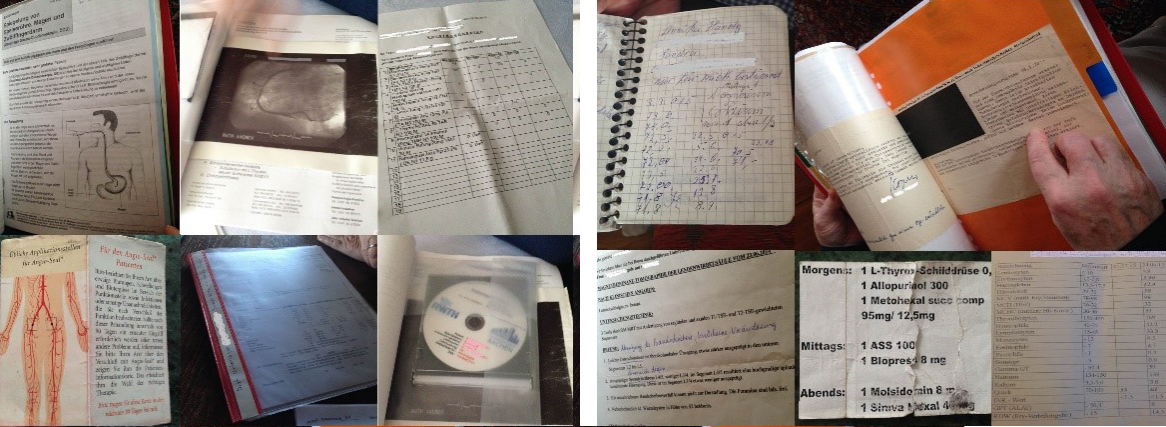
Participant’s documentation of health-related information on paper and digital media.

A total of 9 participants showed diverse tools that they already used to record, keep, and exchange information regarding their health. Accordingly, Figure 1 documents pictures and screenshots. These artifacts included descriptions of surgical procedures; examination results of imaging procedures; tables with results of laboratory tests; folders with personal disease histories (prescriptions, diagnoses, findings, etc); handwritten medication overviews and appointment reminders; and personal diaries with medical data such as blood pressure, pain perception, and behavior.

Some participants clearly showed either an active (ID08, ID10, ID17, and ID18) or a passive (ID08, ID10, ID17, and ID18) information behavior. In contrast, ID06, ID07, ID13, and ID15 represented a mixture of active and passive information behaviors and were classified according to the statement into 1 of the 2 groups. ID03, ID04, and ID16 did not state anything about their information behavior. Participants with active information behavior most frequently conducted research on health issues (prevention) and searched for information about diagnoses and (risk of) examinations. Another important point describing active search was to actively exchange health-related information with the social environment and actively asking doctors (in the form of calls or personal conversations) if uncertainties or questions prevailed.

Participants with passive information behavior required a reminder to go to medical checkups instead of actively remembering or investigating information about it. Their passive information search behavior was reflected in the intake of medication. Here, the doctor played an active role in providing information on the effect and intake and giving further advice on the medication. Passive participants perceived information from the social environment instead of actively using information systems such as the internet to find information. The passive information retrieval process started by observing one’s own symptoms before consulting a doctor. Some participants with passive information search behavior perceived health information as less desirable and irritating for some patients.

### Information-Seeking Behavior Questionnaire Data

Answers to the questions on information-seeking behavior showed that the largest part of the sample (7/26, 27%) used health information to change their health behavior. A total of 23% (6/26) used it to complement professional information either as preparation for a conversation or in addition to it (Table 3).

The results suggest that older adults use 2 to 3 sources to find relevant health information. On the basis of all valid answers, 15% (4/26) use 1 information source, 31% (8/26) use 2 sources, 35% (9/26) use 3 sources, 12% (3/26) use 4 sources, and 8% (2/26) use 5 different sources. All participants find health information on television shows. Here, 85% (22/26) of participants mentioned the German television show Visite (reports on medical topics). Other sources include newspapers and magazines (eg, *Apothekenrundschau*, a magazine distributed free of charge in German pharmacies) and the radio. On the basis of all valid answers, 73% (19/26) indicated that their information sources deliver the information they need, whereas 12% (3/26) said that using their information sources does not lead to the information they want. The other 12% (3/26) indicated that their information sources partly deliver the information they need. The majority of participants (20/26, 77%) were willing to share health-related information with other people. Only 19% (5/26) were unwilling to do so. The following table depicts more about how participants characterized their information acquisition (Table 4).

**Table 3:** Percentages of the different purposes older adults use health information for (N=26).

Use	Participants, n (%)
Generate knowledge	4 (16)
Change health-related behavior	7 (27)
Complement doctors’ information	6 (22)
Making health decisions	2 (6)
Treatment	4 (17)
Exchange experiences	3 (11)

**Table 4:** Information-seeking behavior of the sample (N=26).

CISS^a^-item	Valid answers, n (%)					
	Applicable	Rather applicable	Partially applicable	Rather not applicable	Not applicable	Missing
“I am actively seeking information about (my) health.”	6 (33)	2 (11)	4 (22)	0	6 (33)	8 (30)
“I am looking for information about health or my health rather casually.”	5 (29)	1 (6)	0(0)	1 (6)	11(61)	8 (30)
“I am consciously looking for information about (my) health.”	8 (44)	1 (6)	1 (6)	2 (11)	6 (33)	8 (30)
“I am passively looking for information about (my) health.”	7 (39)	0(0)	3 (17)	0(0)	8 (44)	8 (30)
“I am permanently looking for information about (my) health.”	2 (39)	0(0)	0(0)	1 (6)	15 (83)	8 (30)
“I am a curious person.”	16 (64)	4 (16)	3 (12)	0(0)	2 (8)	1 (4)
“I am willing to take risks.”	7 (28)	3 (12)	4 (16)	3 (12)	8 (32)	1 (4)

^a^CIS: Coping Inventory of Stressful Situations.

### The Influence of Coping Strategies on HIN and HISB Interview Data

#### Task-Oriented Coping Strategy

The information needs of the participants (n=5) from the group with task-oriented coping behavior (T group) are clear and varied. For example, they include the exchange of information between health actors, transparent information on doctors and health insurance billing, and even nonexistent information needs. Thus, one assumption is that T group members are more interested in additional process-relevant organizational information. Information about one’s own health is only needed and exchanged when complaints or symptoms occur. A clear diagnosis, cause, and precaution (eg, cancer precaution) and an exchange of experiences are desired. In addition, participant ID08 stated that information needs are primarily “*dependent on the health situation, and therefore are currently low*.”

Across the entire group, doctors are viewed as the most important source of information, even if *the experiences were not always good*. The information behavior concerning one’s own health is symptom-/illness-related and focused on the doctor. The internet is often mentioned but is critically viewed as a means of obtaining health information. For ID02, the lack of knowledge about a technology constitutes a hurdle for technology use, so that “*as far as health is concerned, I don’t go there (comment: the internet) because I don’t know how it works and I can’t use it*.” ID18’s statement that “*A technical system doesn’t replace the doctor. I mean, it can inform me, but the internet can’t treat me*” reflects again the strong reliance on the doctor and shows that information is tied to treatment and action in general.

#### Avoidance-Oriented Coping Strategy

Only 1 female interviewee was assigned to the group with a pure avoidance-oriented coping strategy. The need for information of the interview partners with an avoidance-oriented coping strategy is comparatively low and relates to drug intake and effect. The doctor stands at the center of information retrieval. He/she has the patients’ complete confidence, and information provided by him/her is not questioned or controlled. Great uncertainty and fear of all other sources exists because of fear of fraud or being taken advantage of, which results from personal experience. The doctor initiates any kind of information behavior. There is no individual drive to gain information. General and personal health information is obtained from a small number of information sources.

#### Task- and Avoidance-Oriented Coping Strategy

Only 1 female interviewee was assigned to this group. Her need for information is similar to the needs of group A: she seeks exclusive information about occurring diseases or complaints that are completely provided by doctors. Similarly, the search for information behavior is only active in the case of complaints and then directed solely at doctors. Information available on television is randomly included in the current personal situation. There is high distrust of all sources of information not related to doctors.

#### Task- and Emotional-Oriented Coping Strategy

Each participant in the TE group demonstrated a high need for information. Compared with the participant in group A, this includes just as much diversity, but in the TE group, it focuses much more on the individual than on the indirectly related organizational processes. Moreover, compared with group A, interest is not linked to a disease or symptom but is generally present. To obtain health-related information and data, fitness trackers, blood pressure monitors, and digital training instructions are used to document and independently influence one’s own health. Doctors are mentioned as the most important source, but “*the different diagnoses which one receives from doctors to one and the same symptom show...that one should remain critical toward doctors*” (ID14). In no other group is supplementing medical information with active, personal information gathering so self-evident: “*I always in-form myself in advance before I go to the doctor*.” Additionally, it is no wonder that, compared with other groups, emotional states play a major role here: “*A personal relationship (to the doctor) is very important*” stated ID17 and described trust to be an important factor by stating that “*I find it pleasant when you can see the person directly, look him in the eye. That creates trust*.”

#### Task-, Emotion-, and Avoidance-Oriented Coping Strategy

Of the 3 interview participants in the TEA group, only 1 woman gave detailed answers to the interview questions. As with the participant in group A, doctors are viewed as responsible for providing information about the participant’s health. However, ID07 stated:

those (doctors) unfortunately often lack the feeling for the context. They lack empathy for the situation of uncertainty in which the patient finds himself.

Here, the method of information transfer is primarily criticized, which does not take sufficient stock of the patient’s emotional world: “*You feel dispatched and inadequately treated*” (ID07). Unlike the TE group and similar to the T and A groups, the required information includes diagnoses and medication information and help to interpret laboratory findings and treatment methods. These are obtained without exception from personal sources of information such as the doctor or pharmacist or, in exceptional cases, from a medically trained relative. The remaining male interviewees of the TEA group indicated that they did not want to know about health or their personal health.

The mixed methods analysis of normalized code frequencies in the separate CISS groups supports the preceding qualitative view. The results should be viewed against the background of the group size (A: n=2, T: n=7, TE: n=9, TA: n=2, and TEA: n=4). Codes concerning information behavior most frequently occurred in the TE group.

In short, it can be stated that qualitative interviews suggest an HIN influenced by individual coping strategies. This matches the results of international researchers and theoretical models [15,17,19,21]. Particularly noticeable in the quantitative analysis of the qualitative data was the influence of the avoidance-oriented coping strategy (group A). People who applied the avoidance strategy entirely or partly had a descriptively much lower HIN. This seems to be intrinsically motivated because satisfaction with the doctor was not necessarily accompanied by an increased HIN. The TE group was the most open to technology use and the collection and interaction of and with its own health-related data. Further investigations could serve to identify factors that explain this observation beyond coping strategies.

### The Influence of Coping Strategies on HIN Questionnaire Data

The score of participants’ satisfaction with information at hand (ie, information need; *D*(26)=0.294; *P*<.001) is significantly different from normality. The numerical scores of each dimension were as follows: task-oriented coping *D*(25)=0.158, *P*=.11; emotion-oriented coping *D*(25)=0.114, *P*=.25; and avoidance-oriented coping *D*(24)=0.193, *P*=.80. The bootstrapping method and bivariate correlation models were applied to investigate the relationship between the scores of individual coping strategies and information need.

**Figure 2:**
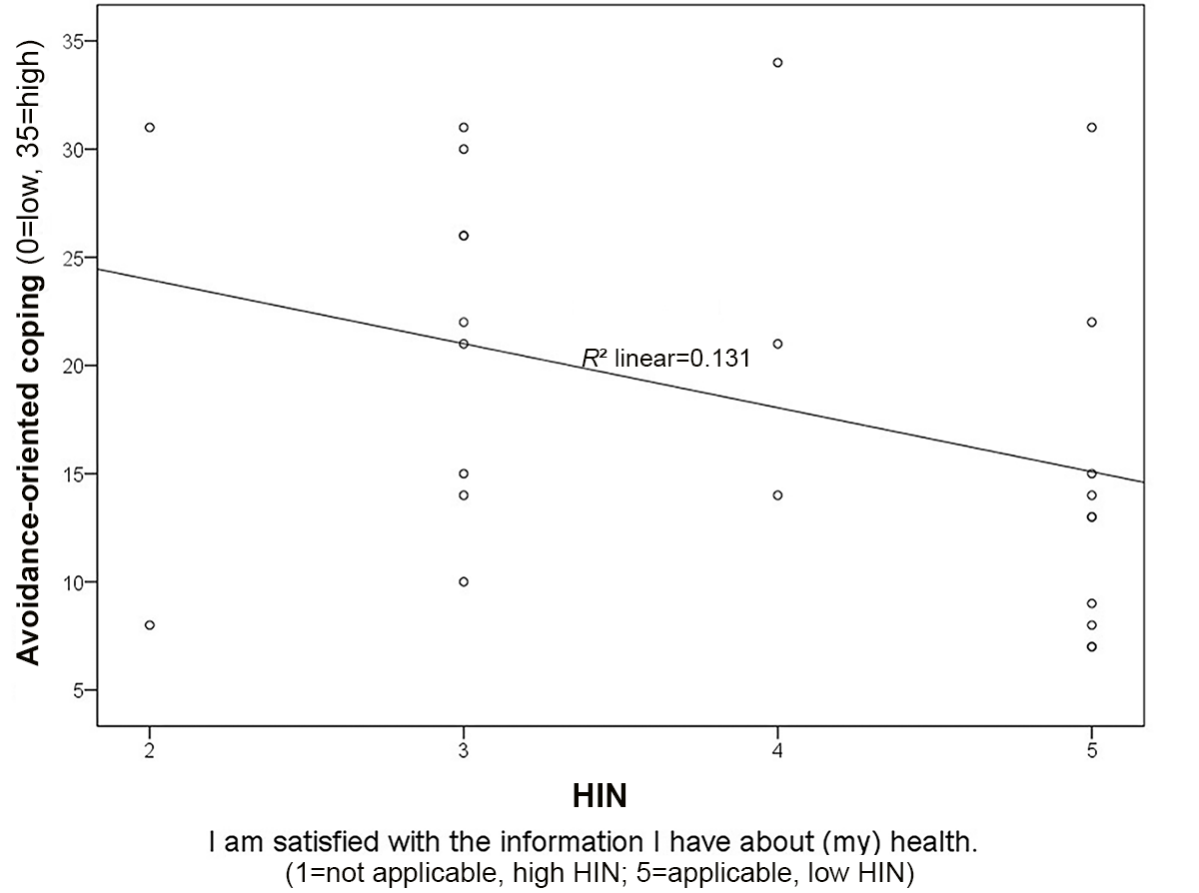
Relationship between the scales of HIN and avoidance-oriented coping. HIN: Health Information Need.

Bias-corrected and accelerated bootstrap 95% CIs (95%, BCa CI) are reported in square brackets. No relationship was found between information need and the task-oriented coping strategy score (*r_s_*=−0.056, 95% BCa CI −0.469 to 0.445; *P*=.79). In addition, no correlation was found between information need and the emotion-oriented coping strategy score (*r_s_*=−0.149, 95% BCa CI −0.532 to 0.260; *P*=.49). However, a trend was found for a negative correlation between the avoidance-oriented scale and information need (*r_s_*=−0.378, 95% BCa CI −0.730 to 0.092; *P*=.05). The more strongly a person is characterized by an avoidance-oriented coping strategy, the lower is the person’s health-related information need (Figure 2).

### The Influence of Coping Strategies on HISB Questionnaire Data

Emotion-oriented coping has a negative relationship with the perceived success of an information source (*r_s_*=−0.607, 95%, BCa CI −0.876 to −0.139; *P*=.20). The more people rely on the emotion-oriented coping strategy, the lower they rated the success of the information they received from the sources they used. The casualness with which a person looks for health-related information is positively related to the success of an information source (*r_s_*=−0.620, 95% BCa CI 0.302 to 0.884; *P*=.01). The more casually a person searches, the higher the person rates the source in terms of success. The consciousness with which a person conducts the information search is positively correlated with the person’s activity level in the search (*r_s_*=0.929, 95% BCa CI 0.839 to 0.982; *P*<.001). The more consciously a person searches, the more active will be the search. The consciousness with which a person conducts the information search is positively correlated with the avoidance-oriented coping strategy (*r_s_*=0.561, *t* 95% BCa CI 0.056 to 0.889; *P*=.03). The more consciously a person searches for health-related information, the higher the scale value of the avoidance-oriented coping strategy is.

## Discussion

### Principal Findings

This study on HINs and HISBs of older adults investigated the general context of data visualizations in a group of 18 adults aged between 50 and 91 years. Interviews on the topic of HINs and HISBs were conducted, transcribed, coded, and qualitatively analyzed. Questionnaires on social demographics and coping strategies served as a basis for comparing qualitative and quantitative results. Essentially, the results indicate a heterogeneous need for information on the part of older people, where one part of the population needs and desires the exchange of personal health data and the other part adopts an attitude of avoidance. There is a need to deal with one’s own health data as a supplement to professional and medical sources of information.

### Discussion of RQs

The first RQ to be answered in this regard was RQ 1: *Which HINs do older adults have?* The results indicate that the health-related information needs of the older people surveyed are not homogeneous. More than half of the participants were satisfied with the information available. According to the definition by Case et al [15], this corresponds to a low need for information. The majority of the interviewees justified the need for health information by stating that they had no health complaints. Another reason provided by the participants in this group was the unsubstantiated assumption that the acquisition and examination of information about one’s own health can lead to the triggering of diseases or an increase in the current pain. This statement is contradictory because a need for preventive, relevant information was often formulated at the same time. This contradiction can point to a need for a more detailed consideration of individual types of health-related information. This study examined the general need for information and revealed that different types of health-related information appear to have different effects on patients and their behavior. In addition to the group that has little or no need for information, there is also a group who makes intensive efforts to gain information about their health. For these people, it is not enough to know what is necessary; rather, they use additional sources of information such as the internet or television to supplement the information they receive from their doctors. For these individuals, information acquisition is considered part of their coping strategy. Only if these patients are sufficiently informed about their illness, do they consider themselves able to make decisions and communicate with doctors.

It was particularly surprising that half of all respondents already collected health data on paper and were using computers. These included notes listing the type and quantity of medication patients carry in their wallets to provide a basis for decision making. Furthermore, doctors had made laboratory results and examination values available to the patients in tabular printouts. Older people had disease histories meticulously collected in folders consisting of examination results, x-rays, medication instructions, and accounts together with detailed visualizations of surgical procedures. Occasionally, participants documented blood pressure, sports activities, and symptoms in a digital form or wrote pain diaries to draw conclusions about causes and adapt their health-related behavior accordingly. The interviewees were among the generation that did not grow up with digital technology. It can, therefore, be assumed that the described number of people digitally documenting their health will increase even further with the growing number of *digital natives*. Here, it is necessary to investigate whether and to what extent the group of information avoiders will play a role in digital health systems that visualize personal health data.

Results regarding health information relevant to older people indicate that more information is needed concerning preventive measures and everyday healthy behavior. Most importantly, there was a lack of a decision-making basis for one’s own behavior. This conclusion is supported by the information regarding the objectives pursued with the collected information. About one-third of the participants stated that they wanted to change their own health behavior according to the information they had collected. The need for information coming directly from the doctor and more intensive communication between doctors and patients reveals the fundamental importance of doctors for older patients. Despite the doctors’ position as the most trustworthy source of information, older people see their lack of time as a barrier to having their information needs met. Most importantly, current billing structures do not allow, for example, detailed clarification of medical terms from the treating physician or receipt of more treatment and diagnosis-specific information from the doctor. Even if digital health systems have the potential to compensate for the doctors’ aforementioned lack of time, the doctors’ acceptance of digital health systems would require a clear billing concept for services provided digitally.

With regard to RQ 2, *How do older adults acquire needed health information?,* the results indicate that health issues and symptoms initiate the information search. Furthermore, if the principal information source—the doctor—does not provide enough information, search activities are initiated. This is in line with the model of information-seeking behavior by Wilson, which states that the failure of one source to provide information motivates search activities. Older adults’ information behavior can thus be considered occasional. Occasional searching could be an alternative explanation for the heterogeneous need for general information. The health status of participants was not explicitly investigated and needs to be considered in future studies on this topic. Furthermore, when it comes to health, the results suggest that the most frequently used and most trustworthy information source is the doctor. These results are consistent with those of age-independent studies. In particular, the older adults attribute medical competence only to the doctor; therefore, they put the decision about a treatment completely in the hands of the doctor. At the same time, similar to the results of the study by Geuter and Weber [7], trust is perceived as a particularly important determinant that arises from personal contact with the treating physician. In addition to the professional competence of the source, the influence of emotional factors on the search for information becomes evident. Besides doctors, television and even the internet are sources of information.

With regard to RQ 3, *How does the coping of older adults relate to their HINs/HISB?,* it can be stated that the qualitative and quantitative results indicate an influence of coping strategy on HIN and HISB. An avoidance-oriented coping behavior especially leads to a lower information need. Assuming that a lower information need results in avoiding information search with technology, avoidance-oriented coping behavior can be considered as a hurdle for health technology and health data visualization use. Further investigations on system design with regard to coping strategies are needed.

### Limitations

The main limitation of this study is its ecological validity. Its cross-sectional design provides insights into HISBs of older adults for a single point in time. Although it closes the research gap of investigating how the specific population of older adults requires information, questions on HINs with respect to ongoing health conditions remain unanswered. As with many studies on the need for health and patient information, the subjective character of this study might be subject to social desirability. Older adults, in particular, often feel the need to conclude from the questions what might be expected from them to adjust their answers accordingly. Future studies on HIN would benefit from controlling this variable. Ecological validity could be improved by investigations within a natural setting, meaning that data from patients’ providers or search engines could be analyzed to triangulate subjective data using objective observational data, interaction, and logfiles.

Furthermore, the study was conducted with participants from Aachen, Germany. Germany’s socioeconomic parameters such as health care, average monthly income, life expectancy, education level, and density of dental care provision match those of other European Union (EU) member countries. Consequently, the data collected should be comparable with those of most of the other EU members, but HINs differ in countries with different economic or cultural backgrounds. At the same time, the results should be subjected to a generalization against the background of sample size and the procedure for acquiring participants.

Another factor that may have influenced the results of the study is that the patients interviewed may have a different understanding of terms in relation to the queried criteria than initially assumed. Against the background of this study, it seems quite probable that the criteria asked for, such as the *health-related information need*, for example, could be understood differently than initially assumed. The patients only answered and assessed these questions on a personal level. In addition, the study participants felt that the question regarding their satisfaction with the information available to them was in part an evaluation of their physician because they understood their physician to be responsible for communicating this information. Their relationship and experience with their physician is thus an influencing factor.

### Conclusions

The results regarding the general need for information identify the need for older people to gain insight into personal health data and to use this as a basis and addition to medical information provided by physicians. This motivates successive investigations on age-differentiated, ergonomic considerations of the visualization of personal health data. However, it should be noted that not every older adult wishes to independently analyze his/her own health data. When provided with health-related data, participants most importantly require support to interpret the data and assess their significance for their personal situation. As the daily use of health-related data puts the disease first, a visualization of data with a direct reference to the disease carries the risk of reduced acceptance, adherence, and use of the corresponding system. In contrast, the need for behavior-changing and preventive measures suggests that data visualizations that allow for conclusions about personal behavior and its correlation with symptoms might positively influence these factors. One unresolved issue in this regard is the extent to which data visualization can increase the motivation of the patient to change a behavior.

This study focused on the need for health information to examine the broad context of digital health systems. Empirical evidence for a correlation between health-related information needs and data visualization/use of technology is lacking and needs to be investigated, especially for the group of older adults. There is also little empirical knowledge about the importance of trust in connection with the visualization of personal health data. Although investigations on factors that influence or generate trust in the data might also be especially relevant for the health care domain, it is still unclear how trust develops in the context of health-related decision making and how corresponding processes proceed or if data visualizations might even increase the user’s acceptance of a digital health system.

Finally, it remains to be clarified whether there is a difference between the information needs of chronically ill and acutely ill people or whether differences in HIN arise predominantly according to observed symptoms or life and care experience. Particularly vulnerable groups (those with Parkinson disease, Alzheimer disease, etc) must be taken into account.

### Implications for Technology Development

The following implications for technology developments were derived from presented results:

1. Digital health technology might be more accepted if its use is recommended and accompanied by the physician.
2. The occurrence of personal symptoms and diagnoses might trigger individual information search behavior.
3. The physician should provide information on illness and medication more effectively, whereas the patient provides information on health-related parameters to the diagnostic process most effectively.
4. Digital systems that can support the patient in everyday documentation of symptoms and complaints to support the diagnostic processes of doctors are required.
5. Patients need support in documenting symptoms and complaints.
6. Cooperation and data exchange of all actors involved in diagnosis and treatment simplifies this for the patient.
7. Comprehensibility and competence of the information source is a key requirement of the patient and should therefore be considered in system development.
8. Adaptive systems for coping strategies are required to address the nonhomogeneous health-related information needs of older adults; therefore, digital health systems must enable patients and users to assess the trustworthiness of information and develop trust.
9. Older adults require diverse types of health-related information and use different methods to acquire information. The planning and development of digital health systems should combine and harmonize different sources of information. Not only user groups with their skills and abilities but also the characteristics of the information sources should be taken into account to effectively coordinate their interaction.
10. Communication strategies implemented in the system that put health rather than illness in the foreground foster acceptance and adherence.

Regarding the design recommendations, it must be considered that these will have to be validated before being actually applicable to system design.

## Appendix

Multimedia Appendix 1 Questionnaires.

## Abbreviations

CISS: Coping Inventory of Stressful Situations
EU: European Union
HIN: health information need
HISB: health information–seeking behavior
RQ: research question
TA: task-avoidance oriented coping
TE: task- emotion oriented coping
TEA: task-emotion-avoidance oriented coping
