# Supplementary material for: The Relationship Between Individual Coping and the Need to Have and Seek Health Information Among Older Adults: Exploratory Mixed Methods Study
Source: JMIR Hum Factors. 2021 Feb 1;8(1):e15858. doi: 10.2196/15858 (PMC7884216; doi:10.2196/15858)
Supplement: Multimedia Appendix 1 [file humanfactors_v8i1e15858_app1.pdf]

Teilnehmer ID_________ Datum____________

Fragebogen: Informationsbedürfnis und –verhalten

Begriffsdefinition:
Gesundheitsbezogene Information: Nach gewissen Regeln strukturierte Daten die eine Bedeutung für die persönliche Gesundheit haben. Ebenso zählen allgemeine gesundheitliche Informationen, Informationen zu bestimmten Krankheiten, Medikamenten, Vorsorge aber auch zu Ärzten, Krankenhäusern und Krankenkassen hierzu.

Persönliche Angaben

1. Geburtsjahr: _________________

2. Höchster Bildungsabschluss: _____________________

3. Ich habe umfangreiche Erfahrungen im Umgang mit Gesundheitsdaten/-informationen?

☐	☐	☐	☐	☐
Trifft zu	trifft eher zu	teils-teils	trifft eher nicht zu	trifft nicht zu

5. In welchem Land wurden Sie geboren?

____

6. In welchem Land haben Sie den Großteil ihres bisherigen Lebens verbracht?

____

7. Wieviel Kinder haben Sie? ____________

Teilnehmer ID_________ Datum____________

8. Welche Berufe haben Sie ausgeübt?

____

____

____

____

Hatten Sie während ihrer beruflichen Laufbahn eine Führungsposition inne? Wenn ja, wie lange

____ Ja, ca. __________ Jahre

____ Nein

9. Beschreiben Sie Ihre Momentane Lebenssituation.
   9.1. Wo und mit wem leben Sie zusammen?

   ____

   ____

   ____

9.2. Treffen Sie selbstständig Entscheidungen in Bezug auf Ihre Gesundheit?

___ Ja ____ Nein ____ teilweise ___ k.A

9.3. Können Sie sich uneingeschränkt innerhalb des Wohnortes bewegen?

___ Ja ____ Nein ____ teilweise ___ k.A#

10. Welche Informationsquellen stehen Ihnen zur Verfügung?

____

____

Teilnehmer ID_________ Datum____________

Fragen der modifizierten Baltimore-Studie (Informationsbedarf urbaner Bewohner)

Abschnitt I

1. **Gesundheit/Krankheit**
   Ich würde Sie bitten sich an die letzten Tagen oder Wochen zurückzuerinnern und Sie bitten mir zu erklären, ob Ihnen eine Situation einfällt, in der Sie nützlich und verlässliche Informationen über Gesundheit/Krankheit in Erfahrung bringen wollten, diese jedoch nur schwer bekommen konnten. Fällt Ihnen dazu etwas ein?

   Wenn die Antwort Nein ist:

1.1. Wir interessieren uns für alle Fragen die sie zu ihrer persönlichen Gesundheit oder hatten?

1.2. Können Sie sich zum Beispiel daran erinnern, dass Sie Hilfe bei Tätigkeiten in Bezug auf ihre Gesundheit benötigten? (*Pause)*

1.2.1. Oder, dass dringend wissen mussten wie man etwas tun musste was in Zusammenhang mit Ihrer oder der allgemeinen Gesundheit oder einer Krankheit stand? (*Pause*)

1.2.2. Oder vielleicht wollten Sie auch nur etwas über ihre Gesundheit oder Gesundheit im Allgemeinen wissen?

1.3. Oder hatten Sie zum Beispiel Schwierigkeiten herauszufinden, wo eine sich eine bestimmte Person, ein bestimmter Ort befindet oder wo sich etwas befindet was Sie für Ihre Gesundheit benötigen? (Pause) Oder haben Sie Informationen über eine Organisation, Firma oder Einrichtung? (Pause)

Wenn die Antwort Ja ist:

1.4. Beschreibung des Problems (Angestrebt wird eine Aufforderung durch Fragen wie:

1.4.1. Welche Informationen benötigten Sie genau?

1.4.2. Was mussten Sie sonst noch dazu wissen?

1.4.3. Können Sie sich an ähnliche Situationen erinnern – eine Situation als Sie es schwierig fanden Informationen zu bekommen die Sie dazu benötigten eine Frage zu Ihrer Gesundheit, zu einer Krankheit zu beantworten?

Teilnehmer ID_________ Datum____________

2. **Vitaldaten**
   Lassen Sie uns für eine Minute über Vitaldaten in Zusammenhang mit Gesundheit im Allgemeinen oder Ihre persönliche Gesundheit sprechen. Andere Menschen mit denen wir gesprochen haben darüber berichtet, dass Sie nicht alle für ihre Gesundheit relevanten Vitaldaten selber erfassen oder verstehen können. Denken Sie an Ihre eigene Gesundheit und damit zusammenhängende Vitaldatenparameter (Herzrate, Blutdruck, Gewicht, täglich verbrauchte und aufgenommene Kalorien, Blutzucker etc.) – fällt Ihnen zu Ihren persönlichen Vitaldaten irgendetwas ein, wozu Sie oder Mitglieder aus Ihrer Familie Fragen zu hätten oder wozu Sie sich Gedanken machen oder Probleme sehen?

   Wenn Nein: zur nächsten Frage
   Wenn Ja: Können Sie mir das Problem näher erläutern? Können Sie genaue Fragestellungen oder Probleme beschreiben?

3. **Einnahme von Medikamenten**
   Heutzutage nehmen viele und insbesondere ältere Menschen regelmäßig Medikamente ein. Manchmal haben Sie Probleme damit die vom Arzt oder Apotheker vorgegebene Einnahme einzuhalten oder die Nebenwirkungen und Wirkungszusammenhänge mit anderen Medikamenten verstehen. Haben Sie persönlich oder Mitglieder Ihrer Familie Fragen, Anliegen oder Probleme zum Thema Einnahme von Medikamenten?

   Wenn Nein: zur nächsten Frage
   Wenn Ja: Können Sie mir das Problem näher erläutern? Können Sie genaue Fragestellungen oder Probleme beschreiben?

4. **Vorsorgemaßnahmen**
   Ab einem gewissen Alter werden von Ärzte und Krankenkassen Vorsorgeunter-suchungen für bestimmte Krankheiten oder zur Erhaltung der Gesundheit vorgeschrieben. Manche Menschen beschreiben Probleme dahingehend, dass sie nicht genau wissen wann welche Vorsorgemaßnahmen getroffen werden sollen. Haben Sie persönlich oder Mitglieder Ihrer Familie Fragen oder Anliegen zu diesem Thema die Sie bisher noch nicht genannt haben?

   Wenn Nein zur nächsten Frage
   Wenn Ja: Können Sie mir das Problem näher erläutern? Können Sie genaue Fragestellungen oder Probleme beschreiben?

5. **Behandlung von Krankheiten**
   Lassen Sie uns noch einmal über Behandlung von Krankheiten im Allgemeinen oder über Ihre persönlichen Krankheiten sprechen – wenn Sie das möchten. Sind Ihnen in letzter Zeit Dinge eingefallen die Sie gerne zu einem bestimmten Krankheitsbild wissen möchten oder haben Sie Probleme bestimmte Informationen zu einer Bestimmten Krankheit zu finden. Fallen Ihnen vielleicht auch Dinge ein die Ihre Familie gerne zu Ihrer Krankheit oder Krankheiten allgemein gerne wissen möchten?

   Wenn Nein zur nächsten Frage
   Wenn Ja: Können Sie mir das Problem näher erläutern? Können Sie genaue Fragestellungen oder Probleme beschreiben?

6. **Zusätzliche Themen**
   Wir haben jetzt Fragen, Interessen und Probleme in den Bereichen Gesundheit, Vitaldaten, Medikamenten, Vorsorgemaßnahmen und bei der Behandlung von Krankheiten gesprochen. Sind bei Ihnen oder bei Mitgliedern Ihrer Familie darüber hinaus noch Fragen oder Anliegen zu den folgenden Themen aufgetaucht? (Liste durchgehen)

   Wenn Nein: ankreuzen

Krankenkassen	__ Nein
Ärzten	__ Nein
Krankenhaus	__ Nein
Pflege	__ Nein

Abschnitt II

Sie haben bereits einige Fragen und Probleme erläutert, die Sie kürzlich in Bezug auf Ihre Gesundheit oder Gesundheit im Allgemeinen hatten (*besprochene Fragestellungen nennen*).

1. Wenn Sie aus diesen die Fragestellung auswählen sollten, von der Sie sagen würden, dass sie die Wichtigste für Sie ist oder von der Sie sagen, dass sie diejenige ist mit der sie sich in letzter Zeit am Meisten beschäftigt haben, welche Frage oder welches Thema wäre das?

2. Und welche würden Sie sagen war die zweitwichtigste gesundheitsbezogene Fragestellung für Sie die Sie sich in der letzten Zeit häufiger gestellt haben?

Ich würde diese Fragestellung/ diesen Punkt gerne mit Ihnen etwas detaillierter besprechen. Lassen Sie uns mit (das erstgenannte Problem) beginnen.

3. Wie lange ist es her, dass Sie sich zum ersten Mal mit dieser Fragestellung befasst haben?

   ________ oder _________ oder ________ oder _______
   # Tage # Wochen # Monate # Jahre

4. Haben Sie versucht Informationen von jemandem darüber zu bekommen?

   Wenn Ja: zu Frage 6.
   Wenn Nein: Aufforderung: Zum Beispiel, haben Sie mit jemandem über die Frage oder das Thema geredet oder haben Sie im Internet oder in der Bibliothek gesucht um eine Antwort auf Ihre Frage zu bekommen?

   Wenn Ja: Gehe zu Frage 7
   Wenn Nein: Gehe zu Frage 6.

5. Denken Sie, dass es jemanden gibt, der über die Informationen verfügt die Sie benötigen?

   Wenn Ja: Wer? Woher wissen Sie, dass die genannte Person diese Informationen haben könnte?

Wenn Nein: gehe zu Abschnitt III.

6. Gibt es einen bestimmten Grund, warum Sie bis jetzt noch nicht versucht haben diese Informationen zu bekommen?

   Wenn Ja: Warum?
   Wenn Nein: gehe zu Abschnitt III.

7. Können Sie mir erklären, wie Sie bei der Informationssuche vorgegangen sind – das bedeutet: wen haben Sie kontaktiert und was haben Sie im Detail getan?

8. Sie haben uns einige Kontaktpersonen genannt, bei denen Sie Informationen zu Ihrer gesundheitsbezogenen Fragestellung erhalten haben. Mit wie vielen Personen haben Sie insgesamt gesprochen?

   8.1. Jetzt würde ich gerne etwas mehr über jeden dieser Kontakte herausfinden, mit dem Sie gesprochen haben.
   8.2. Ist diese Person männlich oder weiblich?
   8.3. Kennen Sie diese Person persönlich?
      8.3.1. Woher kennen Sie diese Person?
      8.3.2. Welchen Beruf hat die Person?
      8.3.3. Wo arbeitet die Person?

Teilnehmer ID_________ Datum____________

Fragen für alle Teilnehmer

a) Jetzt möchte ich mich Ihnen über andere Wege unterhalten, mit denen man an gesundheitsbezogenen Informationen zu Ihren Fragestellungen bekommen kann.

b)

c) Fernsehen	d) Radio	e) Zeitung/Zeitschrift
Haben Sie etwas im Fernsehen gesehen, was ihnen bei der Suche nach gesundheitsbezogenen Informationen weiterhelfen konnte?	Haben Sie im Radio irgendetwas zu Ihrer Fragestellung/dem Problem gehört?	Haben Sie in der Zeitung/einer Zeitschrift irgendetwas zu Ihrer Fragestellung/dem Problem gelesen?
Welches Programm war das Welcher Sender war das?	In welchem Radioprogramm Auf welchem Sender?	In welcher Zeitung? In welcher Art von Artikel?
Was wurde genau über das Problem / die Frage gesagt?	Was wurde genau über das Problem / Frage gesagt?	Was wurde genau über das Problem / Frage gesagt?
War die Information:<br><br>Sehr hilfreich ……………. 1<br>Hilfreich …………………… 2<br>Hilfreich …………………… 3<br>Nicht hilfreich ……………. 4<br>Überhaupt nicht hilfreich….5	War die Information:<br><br>Sehr hilfreich ……………. 1<br>Hilfreich …………………… 2<br>Hilfreich …………………… 3<br>Nicht hilfreich ……………. 4<br>Überhaupt nicht hilfreich….5	War die Information:<br><br>Sehr hilfreich ……………. 1<br>Hilfreich …………………… 2<br>Hilfreich …………………… 3<br>Nicht hilfreich ……………. 4<br>Überhaupt nicht hilfreich….5
Warum haben Sie diese Informationsquelle gewählt?		

Teilnehmer ID_________ Datum____________

f) Internet	g) Smartphone-Anwendung	h) Bücher
Haben Sie etwas im Internet gefunden, was Ihnen weiterhelfen konnte?	Gibt es eine Smartphone Anwendung die Ihnen Informationen zu dem Thema liefert?	Haben Sie in Büchern irgendetwas zu Ihrer Fragestellung/dem Problem gelesen?
Auf welcher Internetseite habe Sie Informationen gefunden? Wer stellt die Internetseite bereit?	Um welche App handelt es sich dabei? Welches Smartphone haben Sie dabei verwendet	In welcher Zeitung? In welcher Art von Artikel?
Was wurde genau über das Problem oder die Frage gesagt	Was wurde genau sagen die Informationen der App aus? W genau half Ihnen die App bei dem Problem?	Was wurde genau über das Problem oder die Frage gesagt
War die Information:<br><br>Sehr hilfreich ……………. 1<br>Hilfreich …………………… 2<br>Hilfreich …………………… 3<br>Nicht hilfreich ……………. 4<br>Überhaupt nicht hilfreich….5	War die Information:<br><br>Sehr hilfreich ……………. 1<br>Hilfreich …………………… 2<br>Hilfreich …………………… 3<br>Nicht hilfreich ……………. 4<br>Überhaupt nicht hilfreich….5	War die Information:<br><br>Sehr hilfreich ……………. 1<br>Hilfreich …………………… 2<br>Hilfreich …………………… 3<br>Nicht hilfreich ……………. 4<br>Überhaupt nicht hilfreich….5

i) Gespräch mit Arzt	j) Gespräch mit Familie	k) Gespräch mit dem Apotheker
Haben Sie durch das Gespräch mit dem Arzt etwas erfahren, dass Ihnen weitergeholfen hat?	Haben Gespräche mit Familienmitgliedern Ihnen Informationen zu dem Thema geliefert?	Haben Sie mit dem Apotheker über das Problem gesprochen?
Mit welchem Arzt haben Sie über das Problem gesprochen?	Mit wem aus der Familie haben Sie genau darüber gesprochen	Mit wem aus der Apotheke haben Sie genau gesprochen?
Was genau hat er über das Problem oder die Frage gesagt	Was hat das Familienmitglied über das Problem gesagt?	Was wurde genau über das Problem oder die Frage gesagt
War die Information:<br><br>Sehr hilfreich ……………. 1<br>Hilfreich …………………… 2<br>Hilfreich …………………… 3<br>Nicht hilfreich ……………. 4<br>Überhaupt nicht hilfreich….5	War die Information:<br><br>Sehr hilfreich ……………. 1<br>Hilfreich …………………… 2<br>Hilfreich …………………… 3<br>Nicht hilfreich ……………. 4<br>Überhaupt nicht hilfreich….5	War die Information:<br><br>Sehr hilfreich ……………. 1<br>Hilfreich …………………… 2<br>Hilfreich …………………… 3<br>Nicht hilfreich ……………. 4<br>Überhaupt nicht hilfreich….5

l) Wir haben uns über unterschiedliche Informationsquellen unterhalten, die Sie genutzt haben um gesundheitsbezogene Informationen zu bekommen. (Nennungen der in 1. Beschriebenen). Welche dieser Quellen war Ihrer Meinung nach am besten geeignet dafür eine Antwort auf Ihre Fragen zu finden?

m) Sind Ihre gesundheitsbezogenen Fragestellungen zu Ihrer Zufriedenheit beantwortet worden oder haben Sie bis zum jetzigen Zeitpunkt eine zufriedenstellende Lösung für das Problem erarbeiten können?

n) Was planen Sie darüber hinaus um die Frage zufriedenstellend beantworten oder das Problem adäquat lösen zu können? (Aufforderung: Fallen Ihnen noch weitere Punkte ein?)

Abschnitt III – Soziales Umfeld

Jetzt möchte ich Ihnen gerne eine paar Fragen zur Ihrer täglichen Interaktion mit anderen Menschen stellen.

1. Bitte denken Sie für einen Moment an Menschen mit denen Sie in der letzten Woche gesprochen oder gesehen haben. Wie viele dieser Menschen zählen zu den folgenden Kategorien:

   1.1. Familie und Verwandte die nicht in Ihrem Haushalt leben Anzahl _____
   1.2. Ihre Nachbarn Anzahl _____
   1.3. Freunde oder nahe Bekannte Anzahl _____
   1.4. Leute mit denen Sie zusammenarbeiten Anzahl _____
   1.5. Leute die nicht zu den oben genannten Gruppen zählen und mit denen ich nur gesprochen haben Anzahl _____

2. Sind Sie Mitglied einer Organisation? Damit sind zum Beispiel kirchliche Gruppen und Vereinigungen, berufliche Assoziationen, Schulorganisationen, Nachbarschaftsgruppen etc. gemeint. Wie nennt sich die Gruppe?

____

Teilnehmer ID_________ Datum____________

Fragebogen zu Wilsons-Modell I. und II.

**Frage**
Ich bin zufrieden mit den Informationen die mir über Gesundheit/mein Gesundheit zur Verfügung stehen.<br>☐ trifft zu ☐ trifft eher zu ☐ teils-teils ☐ trifft eher nich zu ☐ trifft nicht zu
Welche Informationsquellen nutzen Sie um an benötigte gesundheits/-krankheitsbezogene Informationen zu gelangen?<br>____<br>____
Führt die Nutzung dieser Informationsquellen dazu, dass Sie die benötigten Informationen erhalten?<br>____ Ja ____ Nein
Was machen Sie mit den Gefundenen Informationen? Wofür setzen Sie die Informationen ein?

Teilnehmer ID_________ Datum____________

Wollen Sie die Informationen über Gesundheit / ihre Gesundheit mit anderen Menschen teilen?

____ Ja ____ Nein

Begründung: ____________________________

____

____

Wie würde der optimale Informationsaustausch mit diesen Menschen aussehen? Beschreiben Sie kurz.

____

____

____

____

Welche Eigenschaften sollte die Quelle haben aus der Sie Ihre Informationen beziehen?

____

Welche Eigenschaften sollte ein technisches System (z.B. Suchmaschine) haben, wenn Sie es zur Suche verwenden?

____

____

____

Teilnehmer ID__________ Datum_____________

Teilnehmer ID_________ Datum____________

1. Ich suche aktiv nach Informationen über Gesundheit / mein Gesundheit.

☐	☐	☐	☐	☐
trifft zu	trifft eher zu	teils-teils	trifft eher nich zu	trifft nicht zu

2. Ich suche Informationen über Gesundheit / meine Gesundheit ehe beiläufig.

☐	☐	☐	☐	☐
trifft zu	trifft eher zu	teils-teils	trifft eher nich zu	trifft nicht zu

3. Ich suche bewusst nach Informationen über meine Gesundheit.

☐	☐	☐	☐	☐
trifft zu	trifft eher zu	teils-teils	trifft eher nich zu	trifft nicht zu

4. Ich suche passiv nach Informationen über Gesundheit / mein Gesundheit.

☐	☐	☐	☐	☐
trifft zu	trifft eher zu	teils-teils	trifft eher nich zu	trifft nicht zu

5. Ich suche dauerhaft nach Informationen über Gesundheit / mein Gesundheit.

☐	☐	☐	☐	☐
trifft zu	trifft eher zu	teils-teils	trifft eher nich zu	trifft nicht zu

Teilnehmer ID__________ Datum_____________

6. Ich bin ein neugieriger Mensch

☐	☐	☐	☐	☐
trifft zu	trifft eher zu	teils-teils	trifft eher nich zu	trifft nicht zu

7. Ich bin risikofreudig?

☐	☐	☐	☐	☐
trifft zu	trifft eher zu	teils-teils	trifft eher nich zu	trifft nicht zu

1. Wenn medizinische Aufgaben und Tätigkeiten durch digitale Technik und unterstützt werden, spricht man von Telemedizin. Arzt und Patient müssen so nicht an einem Ort sein. Welche medizinischen Aufgaben und Tätigkeiten können Ihrer Meinung nach durch telemedizinische Systeme unterstützt werden? (Nennen Sie so viele Aufgaben wie möglich)

____

____

2. Welche Daten spielen für telemedizinische Anwendungen eine Rolle?

____

____
